# Optimal debulking surgery in ovarian cancer patients: MRI may predict the necessity of rectosigmoid resection

**DOI:** 10.1186/s13244-024-01725-5

**Published:** 2024-06-18

**Authors:** Xiaofang Zhao, Ping Yang, Liu Liu, Yi Li, Yang Huang, Huali Tang, Yin Zhou, Yun Mao

**Affiliations:** Department of Radiology, The First Affiliated Hospital of Chongqing Medical University, Chongqing, China

**Keywords:** Ovarian cancer, Debulking surgery, Bowel resection, MRI, Neoadjuvant chemotherapy

## Abstract

**Objectives:**

To determine whether MRI can predict the necessity of rectosigmoid resection (RR) for optimal debulking surgery (ODS) in ovarian cancer (OC) patients and to compare the predictive accuracy of pre- and post-neoadjuvant chemotherapy (NACT) MRI.

**Methods:**

The MRI of 82 OC were retrospectively analyzed, including six bowel signs (length, transverse axis, thickness, circumference, muscularis involvement, and submucosal edema) and four para-intestinal signs (vaginal, parametrial, ureteral, and sacro-recto-genital septum involvement). The parameters reflecting the degree of muscularis involvement were measured. Patients were divided into non-RR and RR groups based on the operation and postoperative outcomes. The independent predictors of the need for RR were identified by multivariate logistic regression analysis.

**Results:**

Imaging for 82 patients was evaluated (67 without and 15 with NACT). Submucosal edema and muscularis involvement (OR 13.33 and 8.40, respectively) were independent predictors of the need for RR, with sensitivities of 83.3% and 94.4% and specificities of 93.9% and 81.6%, respectively. Among the parameters reflecting the degree of muscularis involvement, circumference ≥ 3/12 had the highest prediction accuracy, increasing the specificity from 81.6% for muscularis involvement only to 98.0%, with only a slight decrease in sensitivity (from 94.4% to 88.9%). The predictive sensitivities of pre-NACT and post-NACT MRI were 100.0% and 12.5%, respectively, and the specificities were 85.7% and 100.0%, respectively.

**Conclusions:**

MRI analysis of rectosigmoid muscularis involvement and its circumference can help predict the necessity of RR in OC patients, and pre-NACT MRI may be more suitable for evaluation.

**Critical relevance statement:**

We analyzed preoperative pelvic MRI in OC patients. Our findings suggest that MRI has predictive potential for identifying patients who require RR to achieve ODS.

**Key Points:**

- The need for RR must be determined to optimize treatment for OC patients.
- Muscularis involvement circumference ≥ 3/12 could help predict RR.
- Pre-NACT MRI may be superior to post-NACT MRI in predicting RR.

**Graphical Abstract:**

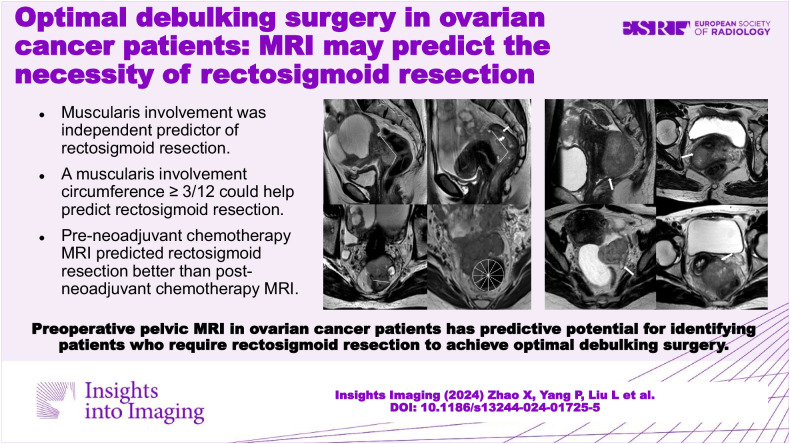

## Introduction

Debulking surgery and platinum-based chemotherapy are the main treatments for ovarian cancer (OC) patients [1]. The residual disease (RD) diameter after surgery is an independent prognostic factor in OC patients. Previous studies have confirmed that patients with optimal debulking surgery (ODS) (i.e., RD ≤ 1 cm) have better progression-free survival and overall survival than patients without ODS. Therefore, the surgical goal for OC is to achieve ODS [2–7]. Invasion of the rectosigmoid colon is the leading cause of RD and constitutes an important factor affecting whether ODS is feasible [8–12]. Rectosigmoid resection (RR) is a radical treatment. In patients with severe rectosigmoid involvement, failure to perform RR may result in recurrence in situ or bowel injury due to forced excision [13, 14]. However, when rectosigmoid involvement is mild, RR is an unnecessary surgical trauma [15]. A colorectal surgeon or a gynecologic oncologist trained in enterectomy is needed to complete RR [16]. Therefore, it is essential to accurately determine whether RR is necessary for OC patients.

However, current methods used to evaluate the rectosigmoid colon in OC patients have limitations. Colonoscopy can observe only the lumen of the bowel; because rectosigmoid invasion progresses from the serosa to the mucosa, the sensitivity of this technique is low [17–19]. Conventional contrast-enhanced CT and CT enterography (CTE) have low sensitivity due to the low soft tissue resolution and rectosigmoid colon collapse that easily obscures the lesions [20–22]. Transvaginal sonography is limited by its inability to observe lesions in the upper rectum and sigmoid [23]. Although diagnostic laparoscopy and exploratory laparotomy are currently the most commonly used evaluation methods, patients may suffer unnecessary trauma [24, 25]. MRI, due to its noninvasive nature and high soft tissue resolution, has become an important preoperative imaging examination for OC patients [26]. Rizzo et al [27] diagnosed rectosigmoid involvement in OC based on axial T2WI and diffusion-weighted imaging (DWI), and the results showed high sensitivity (94.2%) and low specificity (40.0%). Indeed, the assessment of rectosigmoid involvement requires a combination of multiplanar and multiparametric, morphological and functional, and unenhanced and contrast-enhanced sequences. MRI has been widely used in the preoperative evaluation of rectal cancer, but whether it is suitable for the preoperative prediction of RR in OC patients requires further study [28]. Additionally, it is not yet known which is the more appropriate reference image for RR decision-making in patients undergoing neoadjuvant chemotherapy (NACT), pre-NACT MRI, or post-NACT MRI.

This study aimed to determine whether preoperative MRI could accurately predict OC patients who require RR to achieve ODS and to compare the predictive accuracy of pre-NACT MRI and post-NACT MRI in patients receiving NACT.

## Methods

### Study population

The inclusion criteria were patients who underwent debulking surgery and were pathologically confirmed OC (including tubal and primary peritoneal cancers, collectively referred to as OC in our study) at our institution from January 2012 to May 2022. Exclusion criteria: (1) patients without preoperative pelvic MRI were excluded; (2) due to the need to compare the prediction accuracy of pre- and post-NACT MRI, patients who received NACT but lacked pre-NACT baseline MRI and/or post-NACT MRI were excluded; (3) since fat-suppressed T2WI cannot clearly display the structure of the intestinal wall, patients without axial and/or sagittal non-fat-suppressed T2WI were excluded; (4) to avoid affecting the evaluation results due to changes in condition, patients with more than 30 days between the last preoperative MRI examination and surgery were excluded; (5) patients with poor image quality or in whom the cross-section of the affected bowel cannot be visualized were excluded; (6) patients with no lesions in the rectosigmoid colon and no related intestinal operations during surgery were excluded; (7) patients with the Federation International of Gynecology and Obstetrics (FIGO) stage I were excluded; and (8) patients without postoperative follow-up images for at least 1 year were excluded.

### MRI technique

MR examinations were performed using one 1.5-T MRI scanner (Singa HD Excite, GE Healthcare, USA) and three 3.0-T MRI scanners (Singa HD Excite, GE Healthcare, USA; Magnetom Skyra, Siemens Healthineers, Germany; Ingenia, Philips Healthcare, Best, The Netherlands) with pelvic phased-array coils. All MRI protocols included at least axial or sagittal T2-weighted non-fat-suppressed images, axial T1-weighted fat-suppressed or non-fat-suppressed images, axial DWI (b values of 0, 800, and 1000 s/mm^2^), and axial dynamic contrast-enhanced (DCE) T1WI. The contrast agent was Magnevist (Ga-DTPA, Bayer Pharma AG, Germany) with an injection dose of 0.2 mL/kg and an injection flow rate of 2–3 mL/s. Supplemental Table 1 provides the scanning parameters.

### Image analysis

MRI analysis of all OC patients was performed independently on a picture archiving and communication system (Carestream, GCRIS, Shanghai, China) workstation by two radiologists with more than 10 years of experience in abdominal MRI diagnosis (radiologist Y.Z.) and 3 years of experience (radiologist X.F.Z.), who were blinded to previous imaging reports, surgical information, and histopathological information.

**Fig. 1:**
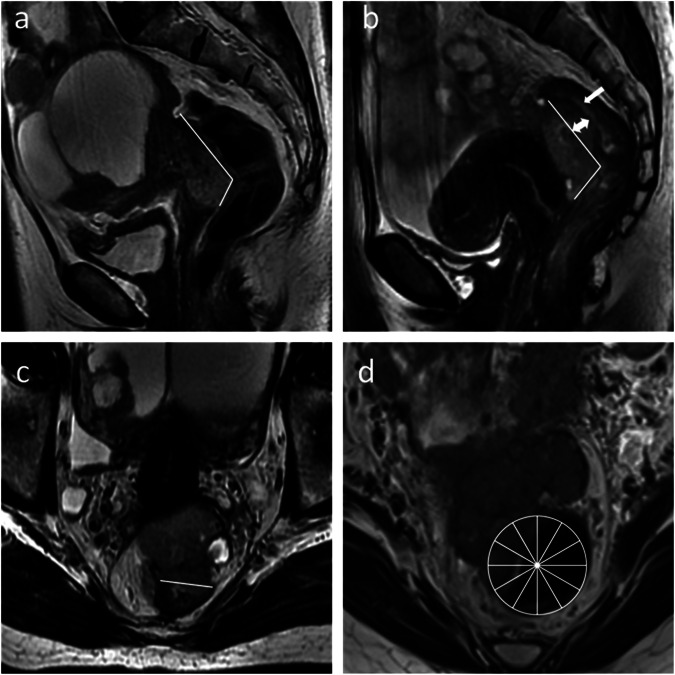
Illustration of bowel MRI signs. **a** Sagittal T2WI shows “minimal” thickening of the serosal layer of the rectosigmoid wall and continuous with the lesions and shows measurements of the length of rectosigmoid involvement (white line). **b** Sagittal T2WI shows the loss of the normal T2-hypointensity of the muscularis in a “fan-shaped” configuration, along with submucosal edema (single arrow), and shows the measurements of length (white line) and thickness (double arrow) of muscularis involvement. **c** Axial T2WI shows the measurement of the transverse axis of muscularis involvement (white line). **d** Axial T2WI shows the rectosigmoid circumference divided into 12 equal parts to evaluate the circumference of muscularis involvement and shows muscularis involvement along 4/12 of the circumference

**Fig. 2:**
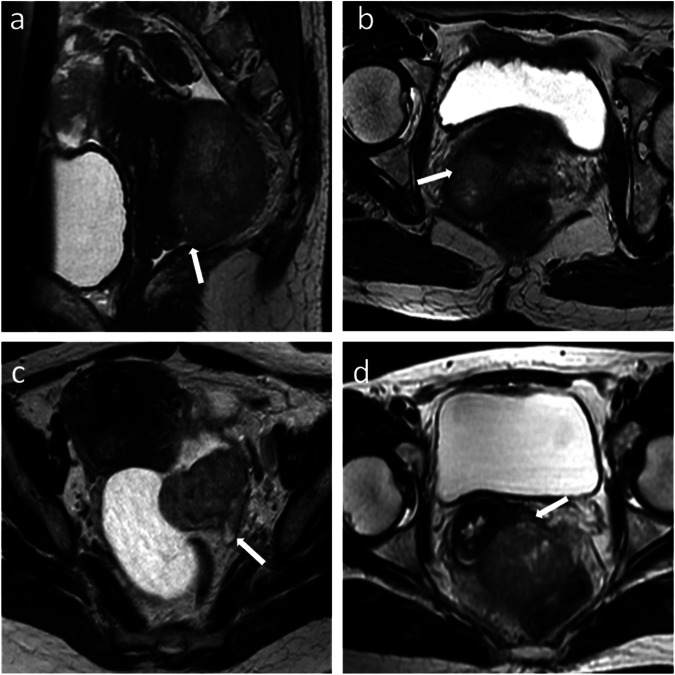
Illustration of para-intestinal MRI signs. **a** Sagittal T2WI shows tumoral nodules extending to the normal T2-hypointense vaginal wall. **b** Axial T2WI shows tumoral nodules extending to the right parametrium. **c** Axial T2WI shows the loss of the fat plane between the lesion and the left ureter with ureteral dilatation. **d** Axial T2WI shows tumoral nodules extending to the left sacro-recto-genital septum with nodular thickening of the left sacro-recto-genital septum

MRI analysis included six bowel signs (length, transverse axis, thickness, circumference of rectosigmoid involvement, muscularis involvement, and submucosal edema) (Fig. 1) and four para-intestinal signs (vaginal, parametrial, ureteral, and sacro-recto-genital septum involvement) (Fig. 2). Axial and sagittal non-fat-suppressed T2WI were the main sequences. The evaluation criteria and procedures were as follows: (1) the lesions were identified on T2WI, DWI, and DCE T1WI. Rectosigmoid involvement was defined as loss of the fat plane between the bowel wall and the lesions, with at least a “minimal” thickening of the serosal layer of the bowel wall and continuous with the lesions [29]. Then, the length, transverse axis, thickness, and circumference of rectosigmoid involvement were measured. The length was measured along the involved bowel wall on sagittal T2WI. Thickness and transverse axis were measured at the deepest and widest point of bowel involvement on axial or sagittal T2WI, respectively. The circumference was scored by dividing the cross-section of the bowel into 12 equal parts. When the bowel involvement was multifocal, the length was the sum of all segments, and the transverse axis, thickness, and circumference were the maximum values. (2) Determined whether muscularis involvement was present. Muscularis involvement was defined as the loss of the fat plane between the bowel wall and the lesions, accompanied by the loss of the normal T2-hypointensity of the muscularis (with or without a “fan-shaped” configuration) [29]. Then, parameters reflecting the degree of muscularis involvement, i.e., the length, transverse axis, thickness, and circumference of muscularis involvement, were measured. (3) Determined whether submucosal edema was present. Submucosal edema was defined as high intensity at the luminal side of the involved bowel wall on T2WI. (4) The evaluation criteria for the four para-intestinal signs were as follows: vaginal involvement manifesting as tumoral nodules extending to the normal T2-hypointense vaginal wall. Parametrial involvement was defined as tumoral nodules extending to the parametrium. Ureteral involvement, either unilateral or bilateral, was defined as loss of the fat plane between the lesion and ureter with ureteral dilatation. Sacro-recto-genital septum involvement, either unilateral or bilateral, was defined as nodular or irregular thickening [30]. (5) For patients undergoing NACT, pre-NACT baseline MRI and the last preoperative MRI were evaluated in the same way as described above.

### Surgical data and grouping criteria

All operations were performed by a gynecologic oncologist experienced in debulking surgery, or a colorectal surgeon experienced in enterectomy if enterectomy was determined. The actual surgical method may not be the best surgical method for achieving ODS. Thus, the rectosigmoid conditions during the operation (including bowel wall injury, lumen stenosis, and RD) and postoperative outcomes (including pathological results and recurrence in situ at least 1 year after surgery) were used as supplementary bases for the classification of actual surgical results. All patients’ clinical data were obtained from the electronic medical record system. RD in this study specifically referred to postoperative RD on the rectosigmoid colon, excluding RD elsewhere. The actual surgical results were divided into eight types, designated type 1–type 8. Then, according to these eight actual surgical results, the patients were divided into a non-RR group and an RR group (Table 1). Among these groups, the actual type 1–type 2 bowel surgery was non-RR, and the postoperative outcomes also confirmed that the ideal operation was non-RR. The actual type 3 bowel surgery was RR, but the postoperative outcomes indicated that the ideal operation was non-RR. The actual type 4–7 bowel surgery was non-RR, but the postoperative outcomes indicated that the ideal operation was RR. The actual type 8 bowel surgery was RR, and the postoperative outcomes also confirmed that the ideal operation was RR.

**Table 1:** Grouping criteria for OC patients

Grouping	Actual surgical results
Non-RR group	Type 1. non-RR + no bowel wall injury + RD ≤ 1 cm + no recurrence in situ at least 1 year after surgery. Type 2. non-RR + no lumen stenosis after the injured bowel wall was repaired + RD ≤ 1 cm + no recurrence in situ at least 1 year after surgery. Type 3. RR + pathological results showed no rectosigmoid involvement.
RR group	Type 4. non-RR + no bowel wall injury + RD ≤ 1 cm + recurrence in situ at least 1 year after surgery. Type 5. non-RR + RD > 1 cm + whether the bowel wall is injured or not. Type 6. non-RR + no lumen stenosis after the injured bowel wall was repaired + RD ≤ 1 cm + recurrence in situ at least 1 year after surgery. Type 7. non-RR + lumen stenosis after the injured bowel wall was repaired + no matter if RD is ≤ 1 cm or > 1 cm. Type 8. RR + pathological results showed rectosigmoid involvement.

*RR* rectosigmoid resection, *RD* residual disease

### Statistical analysis

Statistical analysis was performed with SPSS software (version 25.0; SPSS Inc., Chicago, IL, USA). Categorical variables were expressed as counts and percentages, and comparisons between groups were performed using the chi-square test or Fisher exact probability test. Continuous variables were expressed as mean ± standard deviation or median and interquartile range, and comparisons between groups were performed using Student’s *t*-test or the non-parametric Mann–Whitney *U*-test. The interrater reliability was assessed by calculating intraclass correlation coefficients (ICCs) or weighted kappa coefficients. ICCs of < 0.50, 0.50–0.75, 0.76–0.90, and 0.91–1.00 indicate poor, moderate, good, and excellent reliability, respectively [31]. Kappa values of 0.00–0.20, 0.21–0.40, 0.41–0.60, 0.61–0.80, and 0.81–1.00 indicate slight, fair, moderate, substantial, and almost perfect reliability, respectively [32]. Univariate analysis was performed for all MRI signs with ICC ≥ 0.76 or kappa ≥ 0.81. The image analysis results of senior radiologists were used for subsequent statistical analysis. All the variables with significant differences based on the univariate analysis were calculated by multivariate binary logistic regression. For continuous variables, the Youden index was used to determine the cutoff point that yielded the maximum sensitivity and specificity for predicting the necessity of RR. Predictive ability was evaluated by calculating sensitivity, specificity, positive predictive value (PPV), negative predictive value (NPV), and accuracy. *p* < 0.05 was considered a significant difference.

## Results

### Grouping and basic characteristics

**Fig. 3:**
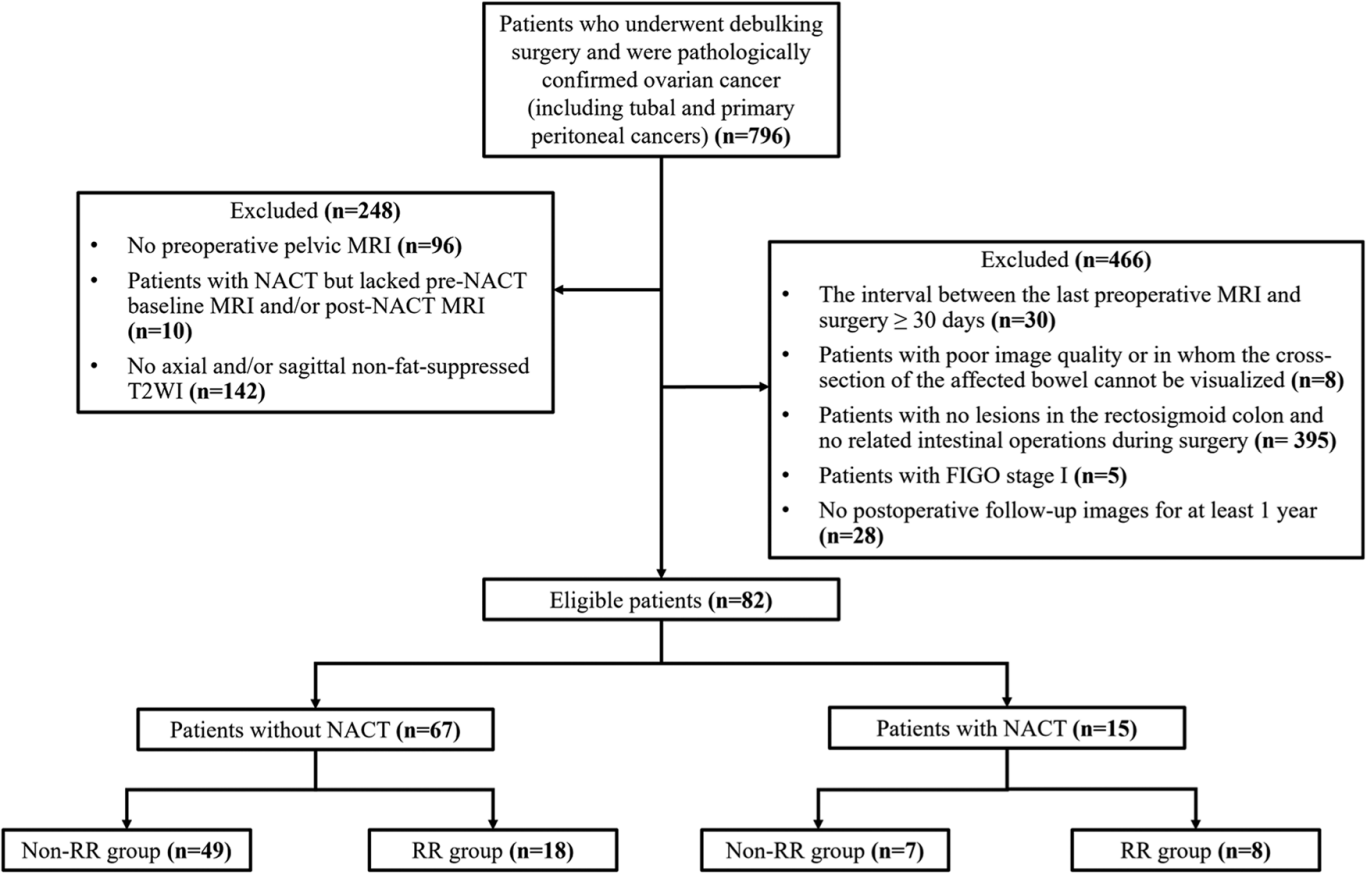
The flow diagram of the patient selection process. NACT, neoadjuvant chemotherapy; FIGO, International Federation of Gynecology and Obstetrics; RR, rectosigmoid resection

A total of 82 OC patients were ultimately eligible for our study cohort (mean age: 53.3 ± 8.8 years; range: 31–75 years), including 67 patients without NACT (49 in the non-RR group and 18 in the RR group) and 15 patients with NACT (seven in the non-RR group and eight in the RR group). In this study, FIGO stages II, III, and IV accounted for 24.4%, 54.9%, and 20.7% of cases, respectively. The distribution of histological types included 68 (82.9%) cases of serous carcinoma, two (2.4%) mucinous carcinoma, six (7.3%) clear cell carcinoma, four (4.9%) endometrioid adenocarcinoma, and two (2.4%) other types. The surgical outcomes revealed 50 (61.0%) cases of type 1, 5 (6.1%) type 2, 1 (1.2%) type 3, 6 (7.3%) type 4, 13 (15.9%) type 5, and 7 (8.5%) type 8, whereas 0 both type 6 and type 7. The flow diagram of the patient selection process is shown in Fig. 3.

### Univariate and multivariate analyses of patients without NACT

The MRI analysis results of patients without NACT are shown in Table 2. All patients with submucosa edema had muscularis involvement. The interrater reliability of all MRI signs was good, excellent, or almost perfect (ICCs from 0.86 to 0.96 and kappa from 0.87 to 1.00).

**Table 2:** MRI analysis results of patients without NACT

MRI signs	Non-RR group, (*n* = 49)	RR group, (*n* = 18)
Length	37.0 (21.0–67.0)	85.5 (34.1, 116.8)
Transverse axis	12.0 (7.0, 16.2)	18.5 (13.5–26.3)
Thickness	0.0 (0.0–0.0)	2.0 (2.0–6.0)
Circumference	2.0 (1.0–3.0)	3.0 (3.0–4.0)
Muscularis involvement	9 (18.4)	17 (94.4)
Length	0.0 (0.0–0.0)	61.5 (35.0–76.3)
Transverse axis	0.0 (0.0–0.0)	17.0 (12.8–22.3)
Thickness	0.0 (0.0–0.0)	4.5 (3.0–7.0)
Circumference	0.0 (0.0–0.0)	3.0 (3.0–4.0)
Submucosal edema	3 (6.1)	15 (83.3)
Vaginal	1 (2.0)	2 (11.1)
Parametrial	2 (4.1)	5 (27.8)
Ureteral	1 (2.0)	0 (0.0)
Sacro-recto-genital septum involvement	5 (10.2)	8 (44.4)

Categorical variables were expressed as counts and percentages, skewed distributed quantitative data are expressed as median and interquartile range

*RR* rectosigmoid resection

In the univariate analysis, all six bowel signs were associated with the necessity of RR (*p* < 0.05), among which submucosal edema and muscularis involvement were strongly positively associated with the necessity of RR (OR 76.67 and 75.56, respectively). Among para-intestinal signs, only parametrial involvement and sacro-recto-genital septum involvement were associated with the necessity of RR (*p* < 0.05). Among the seven patients with parametrial involvement, five had muscularis involvement, and among the 13 patients with sacro-recto-genital septum involvement, nine had muscularis involvement. In the multivariate analysis, submucosal edema (OR 15.00, 95% CI [1.98–113.56], *p* = 0.009) and muscularis involvement (OR 13.33, 95% CI [1.04–170.63], *p* = 0.046) were independent predictors of the need for RR (Table 3). The sensitivity, specificity, PPV, NPV, and accuracy of submucosal edema and muscularis involvement in predicting the necessity of RR were 83.3%, 93.9%, 83.3%, 93.9%, and 91.0% and 94.4%, 81.6%, 65.4%, 97.6%, and 85.1%, respectively.

**Table 3:** Univariate and multivariate analyses of patients without NACT

	Univariate analysis			Multivariate analysis		
MRI signs	OR	95% CI	*p* value	OR	95% CI	*p* value
Length	1.01	(1.00, 1.02)	0.030			
Transverse axis	1.19	(1.07, 1.33)	0.002			
Thickness	6.48	(2.21, 18.97)	0.001			
Circumference	2.67	(1.51, 4.72)	0.001			
Submucosal edema	76.67	(13.96, 420.98)	< 0.001	15.00	(1.98, 113.56)	0.009
Muscularis involvement	75.56	(8.87, 643.79)	< 0.001	13.33	(1.04, 170.63)	0.046
Vaginal	6.00	(0.51, 70.67)	0.355			
Parametrial	9.04	(1.58, 52.07)	0.018			
ureteral	< 0.001	–	1.000^a^			
Sacro-recto-genital septum	7.04	(1.90, 26.13)	0.002			

*OR* odds ratio, *95% CI* 95% confidence interval

^a^ Fisher exact probability test

### Analysis results of the muscularis involvement subgroup without NACT

MRI signs reflecting the degree of muscularis involvement were added to the subgroup analysis of patients without NACT. The interrater reliability of all parameters reflecting the degree of muscularis involvement was good or excellent (ICCs from 0.87 to 0.96).

According to the Youden index, the following cutoff points for measures of muscularis involvement were determined to maximize the probability of ODS and minimize the probability of unnecessary RR: length ≥ 35 mm, transverse axis ≥ 11 mm, thickness ≥ 4 mm, and circumference ≥ 3/12. Among these variables, the circumference ≥ 3/12 predicted the necessity of RR with the highest predictive ability (Table 4). Following the inclusion of circumference ≥ 3/12 of muscularis involvement, the specificity of MRI in predicting the need for RR increased from 81.6% when considering only muscularis involvement to 98.0%. The sensitivity experienced a slight decrease from 94.4% to 88.9%.

**Table 4:** Ability of the degree of muscularis involvement to predict the necessity of RR

MRI signs^a^	Sensitivity	specificity	PPV	NPV	Accuracy
Length ≥ 35 mm	82.4 (14/17)	66.7 (6/9)	82.4 (14/17)	66.7 (6/9)	76.9 (20/26)
Transverse axis ≥ 11 mm	94.1 (16/17)	66.7 (6/9)	84.2 (16/19)	85.7 (6/7)	84.6 (22/26)
Thickness ≥ 4 mm	70.6 (12/17)	88.9 (8/9)	92.3 (12/13)	61.5 (8/13)	76.9 (20/26)
Circumference ≥ 3/12	94.1 (16/17)	88.9 (8/9)	94.1 (16/17)	88.9 (8/9)	92.3 (24/26)

The data in parentheses are the numbers (*n*/*n*) used to calculate the percentage

*PPV* positive predictive value, *NPV* negative predictive value

^a^ All MRI signs are parameters reflecting the degree of muscularis involvement

### Compare the predictive results of pre- and post-NACT MRI

Using a criterion of muscularis involvement circumference ≥ 3/12 to predict the necessity of RR, one false-positive case was identified in the pre-NACT MRI prediction results, while the remaining 14 cases were accurately predicted. In the post-NACT MRI prediction results, there were seven false-negative cases, with the remaining eight cases accurately predicted. The evaluation results of pre- and post-NACT MRI are shown in Table 5.

**Table 5:** The predictive results of pre- and post-NACT MRI for the necessity of RR

		RR group	Non-RR group	Sensitivity	Specificity	PPV	NPV	Accuracy
Pre-NACT MRI	RR group	8	1	100.0%	85.7%	88.9%	85.7%	93.3%
	Non-RR group	0	6					
Post-NACT MRI	RR group	1	0	12.5%	100.0%	100.0%	50.0%	53.3%
	Non-RR group	7	7					

*NACT* neoadjuvant chemotherapy, *RR* rectosigmoid resection, *PPV* positive predictive value, *NPV* negative predictive value

## Discussion

A critical factor in whether an OC patient will achieve ODS is the management of rectosigmoid colon involvement [8–12]. However, a tool that can accurately and noninvasively predict the necessity of RR is still lacking. Our study indicated that preoperative MRI could help identify OC patients who require RR by assessing whether the muscularis of the rectosigmoid colon is involved and, if so, what proportion of the circumference is involved. Furthermore, pre-NACT MRI may provide greater predictive accuracy than post-NACT MRI.

MRI can reliably diagnose rectosigmoid muscularis involvement. This study builds upon the MRI signs of intestinal muscularis involvement identified by Busard et al [29]. This study indicated that muscularis involvement is an independent predictor of RR. Additionally, all patients with submucosal edema had muscularis involvement, and submucosal edema may correspond to nonspecific inflammation caused by deeper involvement of the intestinal wall [29], which manifests itself as T2WI high signal on MRI. Therefore, submucosal edema could serve as an indirect indicator of muscularis involvement, explaining why previous studies have utilized stratification of the intestinal wall at the lesion on CT as a sign of muscularis involvement [33, 34]. Thus, in cases where direct signs of intestinal wall muscularis disruption are unclear, the presence of submucosal edema may aid in diagnosing muscularis involvement and enhance diagnostic sensitivity.

Nevertheless, not all OC patients with muscularis involvement require RR to achieve ODS [14]. Our findings have shown that the extent, especially the circumference, of muscularis involvement is crucial for surgical decisions, which is similar to the results of Rousset et al [35] on endometriosis-related bowel resection. The high predictive accuracy of the circumference of muscularis involvement in this study may be explained by the study of Abrao et al [36]. They found that 89.3% of patients may have mucosal and submucosal involvement when bowel wall involvement exceeds 40% of the circumference. If only tumor excision is performed at this time, it will easily lead to irreparable intestinal wall rupture, necessitating the use of RR. Of course, further pathological verification is required. However, the accuracy of the length of muscularis involvement in predicting RR in this study was relatively low. This may be because repairing the injured small bowel perpendicular to its longitudinal axis is necessary to reduce the risk of luminal stenosis [14, 37]. But the rectosigmoid colon has a larger lumen, making it less likely to develop stenosis even if the repair is not performed perpendicular to the longitudinal axis of the bowel. Regarding para-intestinal signs in this study, parametrial and sacro-recto-genital septum involvement were positively correlated with RR, but they were not independent predictors. The reason may be that most patients with these two MRI signs also had muscularis involvement, making these variables redundant. Therefore, preoperative MRI evaluation of OC patients should include the extent of rectosigmoid muscularis involvement, especially the circumference.

It is not clear whether pre-NACT or post-NACT MRI is a better source of reference images for making decisions regarding RR in OC patients with NACT. In this study, the predictive sensitivity of post-NACT MRI was low. This may be because the signal of inactive fibrosis after NACT may mask the remaining active tumor [38]. It was difficult for MRI to accurately distinguish the remaining active tumors from inactive fibrosis even when enhanced imaging and DWI were used in our study. Recent studies have noted that there may be chemotherapeutic-resistant tumor stem cells in these residual tissues, which are not sensitive to postoperative chemotherapy drugs, and thus easy to cause in situ recurrence [39–42]. Structural disorders of all layers of the bowel wall after NACT also increase the difficulty of MRI evaluation, which is also the reason why T staging via post-NACT MRI for rectal cancer is inaccurate [43]. In this study, there was also one false positive case in the pre-NACT MRI prediction results. Upon analyzing the clinical and imaging data, it was discovered that the patient had isolated metastases confirmed by biopsy in the neck and lung. Following NACT treatment, the lung lesion completely disappeared, and the neck lesion significantly shrank. Although these lesions were not surgically removed, there was no recurrence or enlargement observed during the follow-up period. This suggests that the patient may be sensitive to postoperative chemotherapy drugs, and longer follow-up is necessary to determine the presence of in situ recurrence. But in general, for OC patients receiving NACT, pre-NACT MRI may be more suitable for assessing the necessity of RR.

This retrospective study has several limitations. Firstly, due to its retrospective nature, poorly recorded clinical data in some patients may have impacted the results; therefore, future prospective studies are necessary for further validation of the findings. Secondly, since intestinal recurrence observed by imaging cannot be distinguished from overall tumor recurrence, 1-year follow-up imaging may not accurately reflect postoperative outcomes. Thirdly, the sample size was small; thus, collecting more cases or seeking multicenter cooperation in the future is essential to obtain a larger cohort for further investigation. Fourthly, only MRI analysis was conducted in this study, without comparing the findings with those of other imaging examinations. Future analyses could consider combining MRI with conventional enhanced CT and CTE to improve the accuracy and reliability of results. Finally, the absence of oblique axial T2WI perpendicular to the longitudinal axis of the bowel in this study was partially addressed by excluding patients who could not display a cross-section of the affected bowel.

In conclusion, the presence and circumference of rectosigmoid muscularis involvement on preoperative MRI can help identify OC patients who require RR to achieve ODS. In patients undergoing NACT, pre-NACT MRI may be more appropriate for evaluating the need for RR compared to post-NACT.

### Supplementary information

ELECTRONIC SUPPLEMENTARY MATERIAL

## Data Availability

The datasets used for analyses during the current study are available from the corresponding authors upon reasonable request.

## Abbreviations

CI: Confidence interval
DCE: Dynamic contrast-enhanced
DWI: Diffusion-weighted imaging
FIGO: Federation International of Gynecology and Obstetrics
ICCs: Intraclass correlation coefficients
NACT: Neoadjuvant chemotherapy
NPV: Negative predictive value
OC: Ovarian cancer
ODS: Optimal debulking surgery
OR: Odds ratio
PPV: Positive predictive value
RD: Residual disease
RR: Rectosigmoid resection
