# Supplementary material for: Optimal debulking surgery in ovarian cancer patients: MRI may predict the necessity of rectosigmoid resection
Source: Insights Imaging. 2024 Jun 18;15:145. doi: 10.1186/s13244-024-01725-5 (PMC11183003; doi:10.1186/s13244-024-01725-5)
Supplement: Supplementary file 1 — ELECTRONIC SUPPLEMENTARY MATERIAL [file 13244_2024_1725_MOESM1_ESM.pdf]

Optimal Debulking Surgery in Ovarian Cancer Patients: MRI May Predict the Necessity of Rectosigmoid Resection

ELECTRONIC SUPPLEMENTARY MATERIAL

Supplemental Table 1: The parameters of the MRI scanners

MRI parameters [a]	GE		3.0 T Siemens	3.0 T Phillips
	1.5 T	3.0 T		
Axial T2WI				
Sequence	FRFSE	FRFSE	TSE	TSE
FOV	220-240	220-240	240-260	200-240
ST	4	4	4	3-4
Matrix	320×224	320×320	320×320	348×348
TR	2500-3500	2500-3500	2500-3500	2500-3500
TE	100-105	100-105	88-100	88-100
Sagittal T2WI				
Sequence	FRFSE	FRFSE	TSE	TSE
FOV	200-240	200-240	200-240	200-240
ST	4	4	4	3-4
Matrix	320×224	320×224	320×320	360×243
TR	2500-3500	2500-3500	3000-5000	2500-3500
TE	100-105	100-105	88-100	88-100
Axial T1WI without fat suppression				
Sequence	SE	SE	SE	SE
FOV	200-240	200-240	240-260	200-240
ST	6	6	6	5
Matrix	384×180	384×180	260×320	352×352
TR	560-710	560-762	400-600	540-710
TE	10-14	10-14	10-14	10-14

Axial T1WI with fat suppression				
Sequence	SE	SE	SE	SE
FOV	200-240	200-240	240-260	200-240
ST	6	6	6	5
Matrix	384×180	384×180	260×320	352×352
TR	560-710	560-762	400-600	540-710
TE	10-14	10-14	10-14	10-14
Axial DWI				
Sequence	SSEPI	SSEPI	EPI	TSE
FOV	220-240	220-240	240-260	200-240
ST	5	5	4	4
Matrix	320×256	320×256	256×256	256×256
TR	3200-4100	3200-4100	3200-4800	3200-3800
TE	83-90	83-88	80-88	80-88
Axial DCE T1WI				
Sequence	LAVA	LAVA	VIBE	THRIVE
FOV	220-240	220-240	240-260	200-240
ST	3	3	3	3
Matrix	320×224	320×320	320×320	348×348
TR	3.4-3.5	4.0	3.3	5.4
TE	1.6-1.7	1.9	1.3	0

T2WI, T2-weighted imaging; T1WI, T1-weighted imaging; DWI, diffusion-weighted imaging; DCE, dynamic contrast enhanced; FOV, field of view; ST, section thickness; TR, repetition time; TE, echo time; FRFSE, fast relaxation fast spin–echo; TSE, turbo spin–echo; SE, spin–echo; SSEPI, single-shot echo planar imaging; LAVA, liver acquisition with volume acceleration; VIBE, volume interpolated body examination; THRIVE, T1 high resolution isotropic volume excitation; a, FOV and ST are measured in millimeters, while TR and TE are measured in milliseconds.
